# Gonadal steroids differentially modulate neurotoxicity of HIV and cocaine: testosterone and ICI 182,780 sensitive mechanism

**DOI:** 10.1186/1471-2202-6-40

**Published:** 2005-06-08

**Authors:** Sherie L Kendall, Caroline F Anderson, Avindra Nath, Jadwiga Turchan-Cholewo, Cantey L Land, Charles F Mactutus, Rosemarie M Booze

**Affiliations:** 1 Department of Behavioral Sciences, University of Kentucky, Lexington, Kentucky, USA; 2 Department of Neurology, Johns Hopkins University, Baltimore, MD, USA; 3 Department of Anatomy and Neurobiology, University of Kentucky, Lexington, Kentucky, USA; 4 Behavioral Neuroscience Program, Department of Psychology, University of South Carolina, Columbia, SC, USA

## Abstract

**Background:**

HIV Associated Dementia (HAD) is a common complication of human immunodeficiency virus (HIV) infection that erodes the quality of life for patients and burdens health care providers. Intravenous drug use is a major route of HIV transmission, and drug use is associated with increased HAD. Specific proteins released as a consequence of HIV infection (e.g., gp120, the HIV envelope protein and Tat, the nuclear transactivating protein) have been implicated in the pathogenesis of HAD. In primary cultures of human fetal brain tissue, subtoxic doses of gp120 and Tat are capable of interacting with a physiologically relevant dose of cocaine, to produce a significant synergistic neurotoxicity. Using this model system, the neuroprotective potential of gonadal steroids was investigated.

**Results:**

17β-Estradiol (17β-E_2_), but not 17α-estradiol (17α-E_2_), was protective against this combined neurotoxicity. Progesterone (PROG) afforded limited neuroprotection, as did dihydrotestosterone (DHT). The efficacy of 5α-testosterone (T)-mediated neuroprotection was robust, similar to that provided by 17β-E_2. _In the presence of the specific estrogen receptor (ER) antagonist, ICI-182,780, T's neuroprotection was completely blocked. Thus, T acts through the ER to provide neuroprotection against HIV proteins and cocaine. Interestingly, cholesterol also demonstrated concentration-dependent neuroprotection, possibly attributable to cholesterol's serving as a steroid hormone precursor in neurons.

**Conclusion:**

Collectively, the present data indicate that cocaine has a robust interaction with the HIV proteins gp120 and Tat that produces severe neurotoxicity, and this toxicity can be blocked through pretreatment with ER agonists.

## Background

A particularly devastating complication of HIV infection is a pervasive form of damage to the brain, HAD [1]. The overall incidence of severe HAD is estimated at about 30% of the HIV infected population [2]. However, HAD occurs more often in HIV-positive IV drug users than HIV-positive non-drug users [3-5]. Neuroimaging and autopsy studies demonstrate that the basal ganglia and frontal lobes are preferentially affected by HAD [5,6]. These structures may degenerate with chronic psychostimulant (methamphetamine, cocaine) abuse [7,8], eventually leading to a Parkinson type syndrome [9]. Injection of abused drugs, such as cocaine, has been noted to accelerate the progression of HIV infection to AIDS status and to HAD [8,10-12].

In the United States, cocaine use plays a larger role in HIV transmission to women than it does to men [13]. HIV infected women have lower initial viral loads than men, progress to AIDS status at the same rate as men, yet have higher mortality and lower life expectancy than men [14-16]. How sex differences contribute to the progression to HAD is largely unknown. However, female gender or estrogenic steroids are recognized as protective against several neurological insults, including animal models of ischemia, oxidative stress, and psychostimulant-induced neurotoxicity [17-22] and human neurodegenerative diseases such as Alzheimer's disease [23-26] and Parkinson's disease [27-29]. Tissue culture studies have found that the estrogenic steroid, 17β-E_2_, is protective against neurotoxic HIV proteins [30,31]. Collectively, these gender/hormonal effects suggest that gonadal hormones may play a differential role in effects of drug abuse, HIV infection, and HAD.

Neurotoxic interactions between the HIV proteins, Tat and gp120, and abused psychostimulant drugs have been previously reported [30,32]. More recently, 17β-E_2 _proved neuroprotective *in vitro *[30,32]; however, it is unknown whether this neuroprotection is specifically estrogenic, or also effected by PROG, and whether it contains an androgenic component. Therefore, the aim of the present study was to determine which gonadal steroids provide neuroprotection against the synergistic neurotoxicity of HIV proteins in the presence of cocaine. We investigated the potential for neuroprotection by T, whether this is mediated through an ER mechanism and the potential concentration-dependent neuroprotection by PROG, DHT and cholesterol.

We report here concentration dependent neuroprotection by T mediated through an ICI 182,780-sensitive mechanism. Incomplete neuroprotection at the concentrations (nM) tested was also provided by PROG, DHT, and cholesterol.

## Results

### 17β-, not 17α-Estradiol, protects against HIV proteins plus cocaine synergistic neurotoxicity

**Figure 1:**
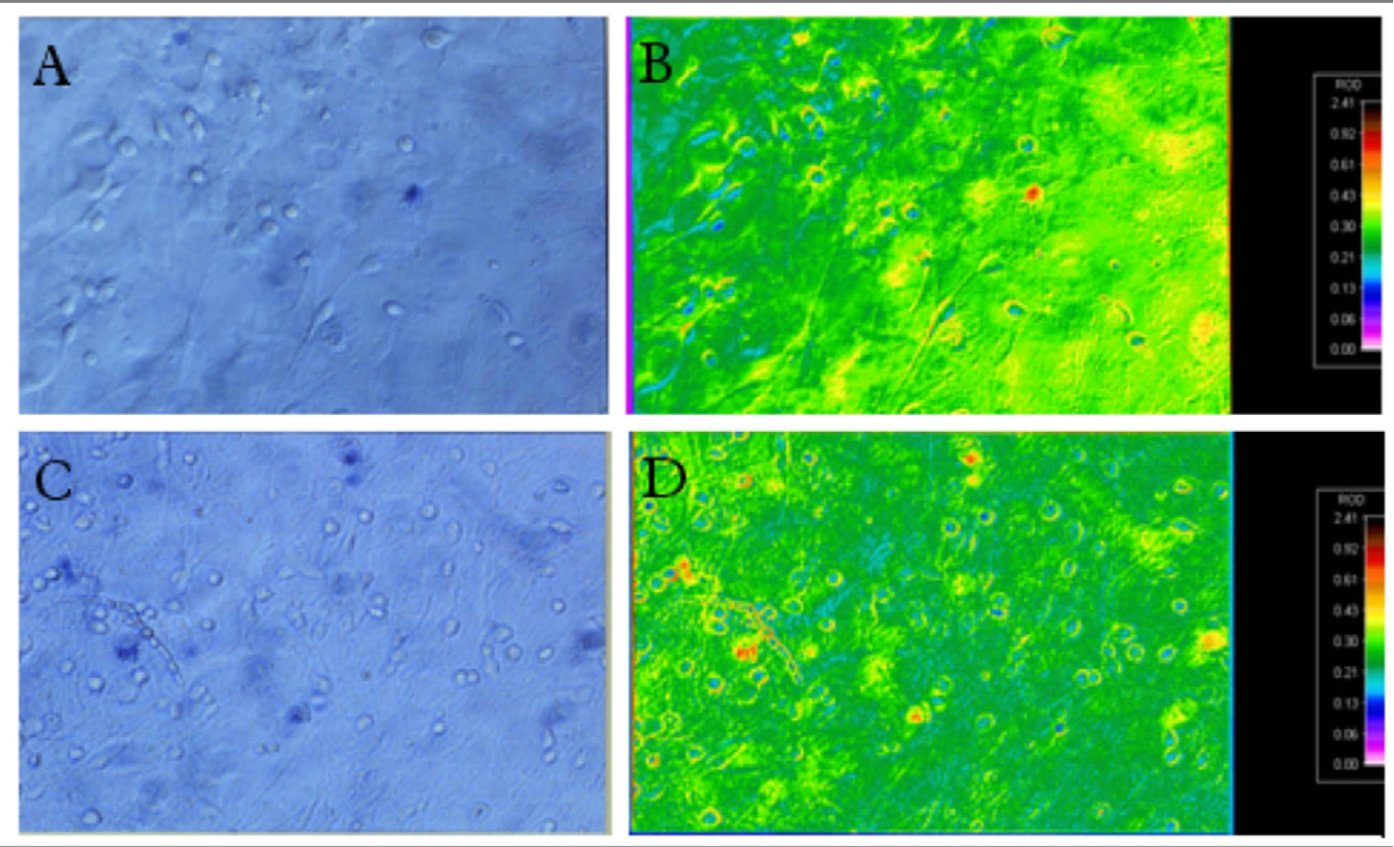
**True and false color photomicrographs of human neurons (A-C). **Control culture after trypan blue exclusion assay in true color (A) and false color (B). Cultures exposed to HIV proteins 40 nM Tat plus 32.5 pM gp120 and 1.6 μM cocaine show synergistic toxicity trypan blue exclusion assay in true color (C) and false color (D).

**Figure 2:**
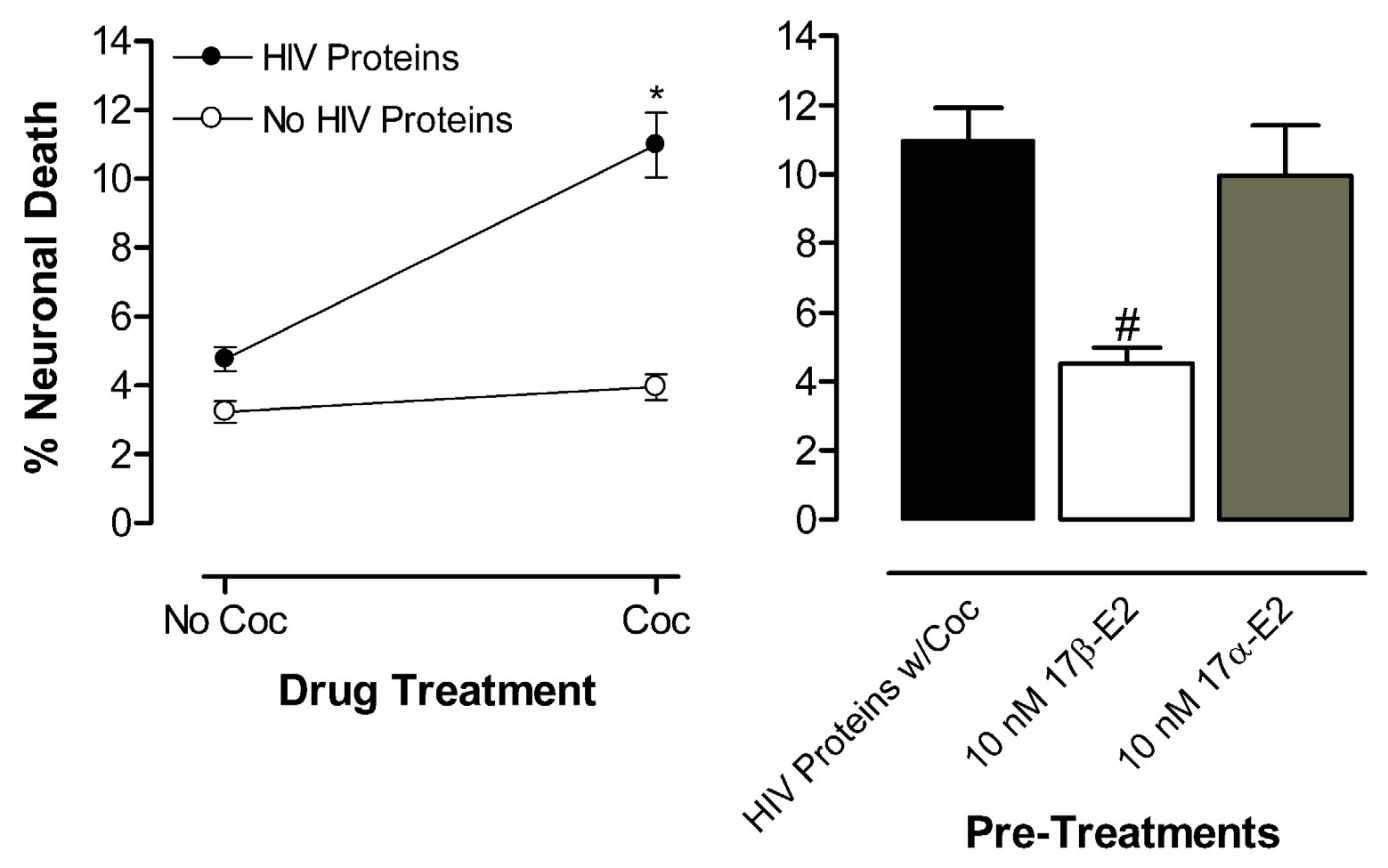
**17β-, not 17α-E_2_, protects human fetal neurons against the synergistically toxic of incubation with Tat [40 nM] plus gp120 [32.5 pM] (HIV Proteins) plus cocaine (Coc) [1.6 μM]. **Control conditions of incubation with Locke's Buffer vehicle or the HIV proteins or Coc only produced similar neuronal death. **(Left Panel) **Significant interaction of the HIV proteins with Coc, as illustrated by the lines diverging from parallel, produced toxic synergism (*p < 0.0008). **(Right Panel) **Significant neuroprotection by 17β-E2 against HIV proteins w/ Coc toxic synergism (#p < 0.0001).

Clear and robust synergistic neurotoxicity of the HIV proteins Tat and gp 120 was repeatedly observed when combined with a physiologically relevant dose of cocaine (Figs. 1C, 1D, 2 Left Panel, 3 Left Panel, 4 Top Panel, 5 Top Panel). False color visualization of the intensity of trypan blue within neurons verified the assay for cell death and demonstrated the synergistic toxicity of cocaine with the HIV proteins (Fig. 1). Furthermore, this neurotoxicity is precluded by pretreatment with 10 nM dose of 17β-E_2_, but not with 17α-E_2_. Neither the HIV proteins, Tat plus gp120, nor cocaine alone were more toxic than the Locke's buffer control; however, in combination they produced synergistic neurotoxicity (Fig. 2). The ANOVA confirmed the presence of a significant interaction of the HIV proteins with cocaine (*F*_(1,24) _= 15.38, *p *< 0.0008; n = 6 each point) (Fig. 2 Left Panel). The presence of this significant neurotoxic effect was demonstrated in every experiment at p < 0.005. The stereoisomers of estradiol demonstrated a significant treatment effect against this toxicity (*F*_(2,15) _= 6.95, *p *< 0.007). The 17β-stereoisomer of estradiol demonstrated significant neuroprotection (*F*_(1,24) _= 24.71, *p *< 0.0001; n = at least 3 each point). The percent neuronal death with 17α-E_2 _treatment was not significantly different from the synergistic toxicity control (Fig. 2 Right Panel).

DMSO at 10 nM (0.1%) served as the solvent for 17α-E_2 _and for ICI-182,780; therefore, it was tested for toxicity and was not significantly different from Locke's buffer incubation (data not shown). β-Cyclodextrin (100 nM) served as an encapsulating carrier to enhance solubility for the steroids 17β-E_2_, T, PROG and cholesterol and was therefore tested for neuroprotection. When coincubated with the toxic combination of HIV proteins plus cocaine, this carrier also had no significant effect on cell death (data not shown).

### Progesterone provides partial neuroprotection against HIV proteins plus cocaine synergistic neurotoxicity

**Figure 3:**
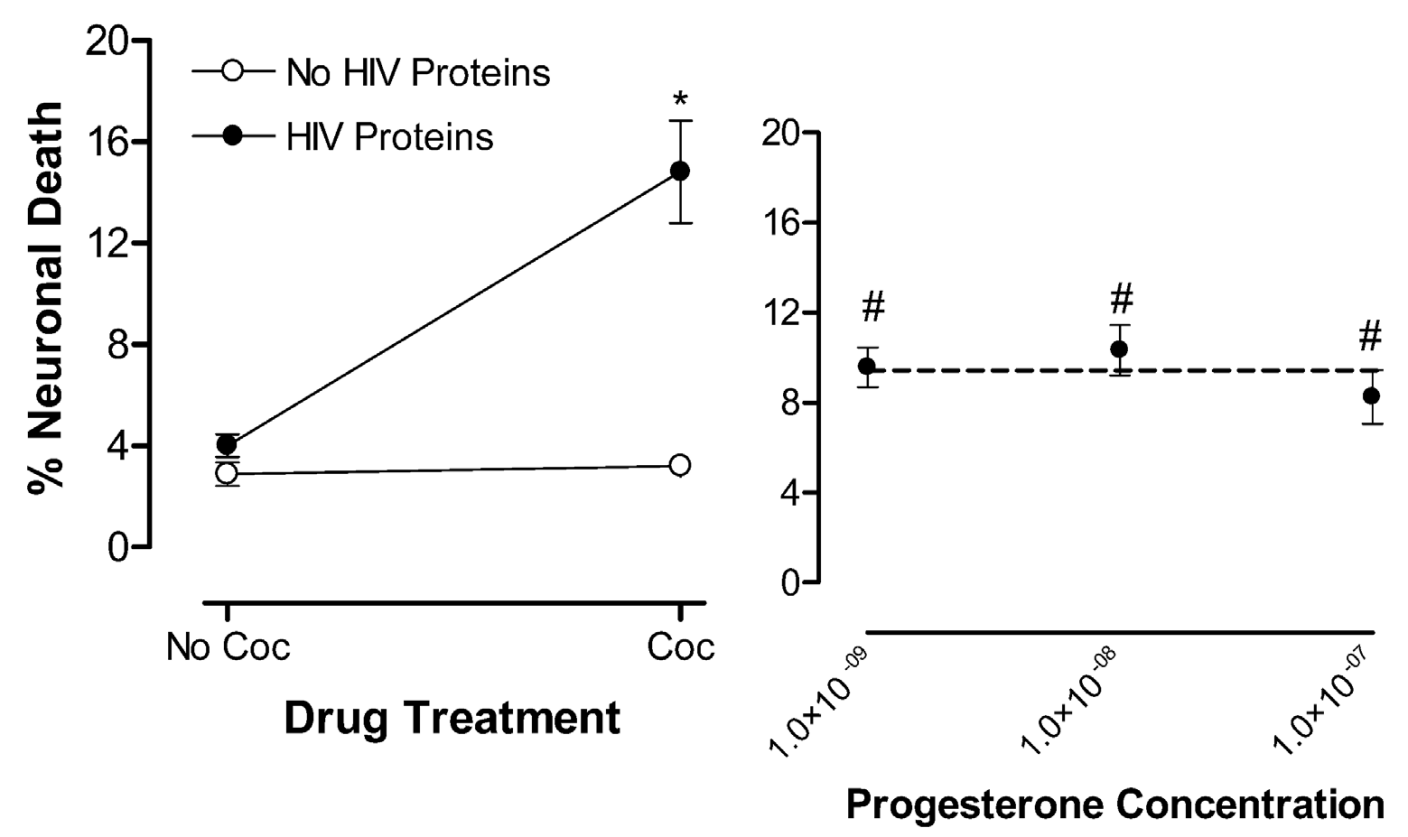
**PROG [M] is partially neuroprotective against synergistic neurotoxicity of HIV proteins with cocaine (Coc). **Control conditions of incubation with Locke's Buffer vehicle or the HIV proteins or Coc only produced similar neuronal death. (**Left Panel**) Significant interaction of the HIV proteins with Coc, as illustrated by the lines diverging from parallel, produced toxic synergism (*p < 0.0044). (**Right Panel**) PROG provided partial neuroprotection against HIV proteins w/ Coc toxic synergism at all concentrations tested (#*p *< 0.0025) however, the neuroprotection was not concentration dependent.

To study the effect of PROG on HIV protein and cocaine synergistic toxicity we treated cultures with 1 nM, 10 nM, and 100 nM doses of PROG prior to adding the toxic combination of HIV proteins and cocaine (Fig. 3 Right Panel). PROG demonstrated significant neuroprotection (*F*_(1,28) _= 11.03, *p *< 0.0025; n = 5 each point). There was no concentration-dependent effect on percent neuronal death (*F*_(1,28) _< 1.0). Further, the neuroprotection was incomplete at these concentrations as the magnitude of cell death was significantly greater than that observed with Locke's Buffer control (*ps *< 0.01 for all concentrations tested). Fig. 3 Left Panel shows the significant interaction of the HIV proteins with cocaine confirming the presence of synergistic neurotoxicity (*F*_(1,16) _= 10.97, *p *< 0.0044; n = 5 each point;).

### Testosterone completely protects against HIV proteins plus cocaine synergistic neurotoxicity

**Figure 4:**
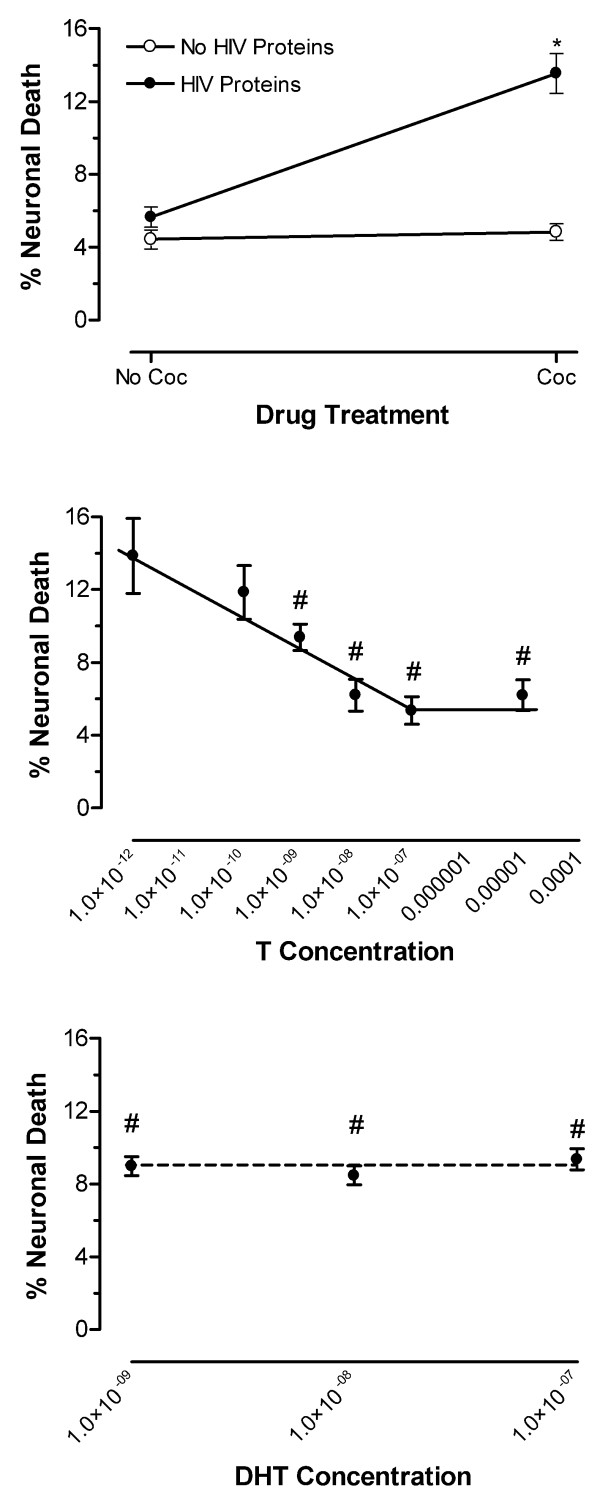
**T [M] provides concentration-dependent neuroprotection against synergistic neurotoxicity of HIV proteins with cocaine (Coc); DHT's [M] neuroprotection is limited. **Control conditions of incubation with Locke's Buffer vehicle or the HIV proteins or Coc only produced similar neuronal death. (**Top Panel**) Significant interaction of the HIV proteins with Coc, as illustrated by the lines diverging from parallel, produced toxic synergism (*p < 0.0087) (**Middle Panel**). T provides significant concentration-dependent neuroprotection against HIV proteins w/ Coc toxic synergism (#*p *< 0.0001). (**Bottom Panel**). DHT mediated partial protection against synergistic neurotoxicity of HIV proteins w/ Coc (#*p *< 0.023); however, the neuroprotection was not concentration dependent.

Concentrations of 1 pM, 100 pM, 1 nM, 10 nM, 100 nM and 10 μM of T were tested against the synergistically neurotoxic combination of HIV proteins and cocaine (Fig. 4). A significant interaction of the HIV proteins with cocaine (*F*_(1,20) _= 8.47, *p *< 0.0087; n = at least 6 each point) confirmed the presence of synergistic neurotoxicity (Fig. 4 Top Panel). T treatment provided significant neuroprotection (*F*_(1,57) _= 12.71, *p *< 0.0007; n = at least 4 each point). Specifically, there was a concentration-dependent effect of T with a significant linear decrease in percent neuronal death as a function of increasing dose (*F*_(1,57) _= 22.56, *p *< 0.0001) (Fig. 4 Middle Panel). The neuroprotective effect of T was characterized against the negative control of Locke's buffer vehicle and the positive control of the synergistically toxic combination of HIV proteins with cocaine. A ceiling effect of maximal neuroprotection at T concentrations of 10 nM or greater, was noted; magnitude of cell death was not significantly different from incubation with Locke's buffer vehicle. A floor effect was identified at the lowest concentrations tested, 100 pM or less, with the magnitude of cell death not significantly different from that observed by the toxic synergism of the HIV proteins with cocaine.

### Dihydrotestosterone provides limited neuroprotection against HIV proteins plus cocaine synergistic neurotoxicity

DHT, the androgen receptor (AR) active metabolite of T, was tested for neuroprotection in the linear range of the T concentration-effect curve, 1 nM, 10 nM and 100 nM, against the toxic synergism of HIV proteins with cocaine (Fig. 4). DHT treatment was confirmed to provide significant neuroprotection (*F*_(1,57) _= 10.66, *p *< 0.002; n = at least 4 each point). However, there was no concentration-dependent effect on percent neuronal death (*F*_(1,57) _<1.0). Further, the neuroprotection was incomplete across these concentrations as the magnitude of cell death was significantly greater than that observed with Locke's Buffer control (*F*_(1,57) _= 6.72, *p *< 0.012) (Fig. 4 Bottom Panel). Because the DHT treatments were run in parallel with the T concentration effects, the control values are the same for both sets of treatments. That is, the presence of synergistic neurotoxicity was indicated by the same significant interaction of the HIV proteins with cocaine (*F*_(1,20) _= 8.47, *p *< 0.0087) as it was for the T treatments (Fig. 4 Top Panel). Ethanol at 10 nM (0.1%) served as the solvent for DHT; therefore, it was tested for toxicity and was found not to differ significantly from Locke's buffer incubation (data not shown).

### ICI-182,780 blocks testosterone's neuroprotection against HIV proteins plus cocaine synergistic neurotoxicity

**Figure 5:**
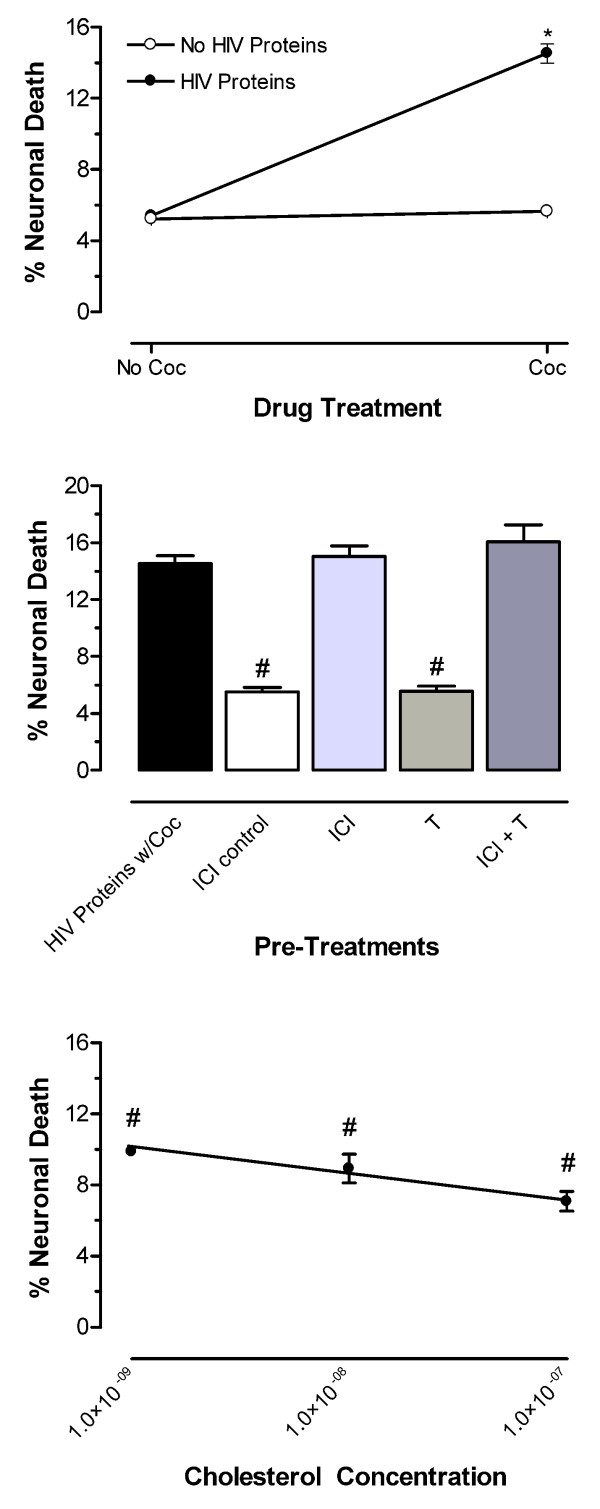
ER mediation of T's protection against synergistic neurotoxicity of HIV proteins with cocaine (Coc); cholesterol's [M] neuroprotection is incomplete. Control conditions of incubation with Locke's Buffer vehicle or the HIV proteins or Coc only produced similar neuronal death. (**Top Panel**) Significant interaction of the HIV proteins with Coc, as illustrated by the lines diverging from parallel, produces toxic synergism (*p < 0.0001. (**Middle Panel**) The ER specific antagonist, ICI-182,780 (ICI), completely blocked T-mediated neuroprotection. ICI's toxicity and T's neuroprotection did not differ from vehicle control (#*p *< 0.0001). (**Bottom Panel**) Cholesterol produced significant, concentration-dependent protection against synergistic neurotoxicity of HIV proteins w/ Coc (#*p *< 0.0001); however full protection was demonstrated only at 100 nM.

The cultures were pretreated with the specific ER antagonist, ICI-182,780 at 100 nM before addition of T (10 nM) then challenged with the HIV proteins plus cocaine synergistic toxicity (Fig. 5). A significant interaction of ICI-182,780 with T was observed (*F*_(1,22) _= 33.71, *p *< 0.0001; n = at least 6 each point). Importantly, the ICI compound was not more toxic to the cultures than was Locke's buffer; nor was it neuroprotective against the toxic challenge. T at 10 nM provided neuroprotection (*F*_(1,65) _= 121.26, *p *< 0.0001), which was not significantly different from incubation with Locke's buffer vehicle alone. This neuroprotective effect of T was fully blocked by pretreatment of the cultures with ICI-182,780 (100 nM) (Fig. 5 Middle Panel). A significant interaction of the HIV proteins with cocaine indicated the presence of synergistic neurotoxicity (*F*_(1,28) _= 61.04, *p *< 0.0001; n = 8 each point) (Fig. 5 Top Panel).

### Cholesterol provides concentration dependent neuroprotection against HIV proteins plus cocaine synergistic neurotoxicity

Cholesterol was added to the cultures at concentrations of 1 nM, 10 nM, and 100 nM prior to treatment with the synergistic toxic combination of HIV proteins plus cocaine (Fig. 5). Significant neuroprotection by cholesterol against this synergistic toxicity was observed (*F*_(1,65) _= 55.74, *p *< 0.0001; n = at least 3 each point) (Fig. 5 Bottom Panel). There was a concentration-dependent effect of cholesterol with a significant linear decrease in percent neuronal death as concentration increased (F_(1,65) _= 4.4042.73, *p *< 0.0398). The neuroprotection afforded by the high dose (100 nM) of cholesterol was asymptotic to a ceiling effect; the magnitude of cell death was not significantly different from that observed by Locke's buffer incubation. Because the cholesterol treatments were performed concurrently with the ICI-182,780 treatments, the control values are the same for both sets of treatments. The presence of synergistic neurotoxicity was indicated by the same significant interaction of the HIV proteins with cocaine as it was for the ICI-182,780 treatments (*F*_(1,28) _= 61.04, *p *< 0.0001) (Fig. 5 Top Panel).

## Discussion

Neuroprotection is clearly afforded by physiological concentrations of 17β-E_2 _in this human fetal neuronal model of gp120 and Tat HIV protein neurotoxic synergism with cocaine [30]. In the current studies, we have replicated and extended these findings to examine the ability of other gonadal steroids to provide neuroprotection against this combined insult. We now show robust neuroprotection mediated through the ER is provided by T while PROG, DHT and cholesterol provide only partial or incomplete neuroprotection in the same concentration range.

There are at least two mechanisms for the neuroprotective actions of estradiol: receptor mediated cellular events; and non-receptor mediated anti-oxidant effects. Stereo-specific neuroprotection by estradiol (Fig. 2) suggests a receptor-mediated mechanism rather than global scavenging of reactive oxygen species (ROS) by the phenolic ring. Both of the estradiol stereoisomers, but not PROG, T, DHT, nor cholesterol contain the phenolic moiety of estradiol. In contradiction to our results, neuroprotection by neutralization of ROS would be exhibited by both estradiol isomers but not the other steroid compounds tested. Moreover, the phenolic ring's efficacy in quenching ROS usually requires higher concentrations than needed for receptor-mediated activity [33-35]. Our data suggest that there is a specific ER modulation of the neurotoxic cascade preventing neural death, rather than nonspecific scavenging of ROS by estrogens.

In these studies, the neurons were exposed to estrogens for 15 hours. This time course (15-hour incubation) does not preclude estrogen-mediated genomic activity; however, estrogens are known to act through both genomic and non-genomic pathways [36,37]. Nevertheless, the lack of effect by the transcriptionally inactive stereoisomer of estradiol (17α-E_2_) provides evidence for a genomic neuroprotective mechanism. Neuroprotection by 17α-E_2 _has been reported in human neuronal cultures [38,39]. This neuroprotection, which was not seen in our model, was realized in some non-genomic manner such as activation of signal transduction. Research reports also support three other non-genomic actions of steroids that may protect neurons from toxic insults [40]: 1) an indirect effect due to structural and functional perturbation of membrane properties by the intercalation of the cholesterol moiety comprising the steroids; 2) a direct activation of membrane-bound steroid specific protein recognition sites; and 3) modulation of classical neurotransmitter receptor activity. Furthermore, neuroprotection conferred by an individual agent may not be attributable to a single mode of action. All steroid hormones, including 17α-E_2_, might be expected to modulate neurotransmission by altering the relative steroidal composition of the neuronal membrane. Since 17α-E_2 _failed to show neuroprotection, these nonspecific membrane effects are ruled out, but activity at a membrane receptor or modulation of neurotransmitter activity is not. We tested six steroids (the two estradiol stereoisomers, PROG, T, DHT and cholesterol) at physiologically relevant nanomolar (nM) concentrations against the synergistic neurotoxic challenge to delineate neuroprotection by steroids in this model of HAD, while considering that each steroid may operate by different as well as pleiotropic means.

Treatments with PROG may be neuroprotective [41-43]. The neuroprotective capability of PROG in the physiological range [44] was also exhibited against our neurotoxic challenge (Fig. 3). Although PROG's neuroprotection was significant, it was incomplete in the nM concentration range tested, as PROG was significantly different from the negative control (i.e., relative to incubation in Locke's buffer). The linear regression analysis confirmed the significance of the neuroprotection. However, PROG as well as some of its metabolites are known to act both as agonists at the intracellular and membrane PROG receptors and as allosteric modulators of some neurotransmission [45].

In contrast, T (10 nM) produced neuroprotection of a similar magnitude to that achieved by 17β-E_2 _(Fig. 3). A log plot of the data (Fig. 4 Middle Panel) describes a linear function between 100 pM and 10 nM. No protection is seen at 1 pM, whereas concentrations above 100 nM show maximal protection, thus establishing the EC_50 _for the neuroprotective effect of T between 1 pM and 100 nM. The pharmacokinetics of a concentration-dependent effect are consistent with a receptor-mediated mechanism of neuroprotection. Because T can be aromatized to estradiol by P450 aromatase expressed by astrocytes [46] and by neurons [47] in rat brain, it is not entirely surprising to see a robust neuroprotective effect of T, as was seen with 17β-E_2_. Such neuroprotection could be produced through activation of the ER. Therefore, the question remained whether an androgenic component to the presumptive steroid receptor-mediated neuroprotection is operating.

T is not specific in identifying the receptor mediating its effects because it is the prohormone for both DHT, the principal ligand at the AR, and 17β-E_2_, the principal ligand at the ER. DHT is a useful androgen for evaluating the relative contributions of AR and ER-mediated neuroprotective effects because it does not serve as an agonist at the ER nor can it be converted to estradiol. Our toxic challenge test results with DHT suggest that the AR may mediate neuroprotection, but do not exclude the possibility of effects by other means (Fig. 4 Bottom Panel). To clarify whether T was acting at the AR or the ER, we used the specific ER antagonist, ICI-182,780, while treating the cultures with T and the toxic combination of HIV proteins and cocaine. The ER antagonist completely blocked T's neuroprotection thus demonstrating that the effect is mediated through the ER (Fig. 5 Middle Panel) at physiological concentrations [48,49]. If an AR-mediated component were active in T's neuroprotection it would have been revealed when T was tested while the ER was pharmacologically blocked by ICI-182,780. Therefore, we conclude that T's neuroprotection is mediated through the ER with no portion attributable to AR activation or to non-genomic pathways.

As a control for general steroidal structural and receptor activation we tested cholesterol, which is known to modulate membrane fluidity but is neurally inactive. We saw that cholesterol's concentration-dependent neuroprotection against synergistic toxicity of the HIV proteins with cocaine is more efficacious than that of PROG and DHT (Fig. 5 Bottom Panel) in that 100 nM cholesterol was fully neuroprotective whereas the same dose of PROG or DHT was not. However, cholesterol is less potent than the gonadal steroids, 17β-E_2 _and T in that 10 nM cholesterol was not fully neuroprotective, whereas the same dose of the steroids was. The mechanism of this protection is interesting because the cholesterol moiety is common to all steroids, but 17α-E_2 _was not neuroprotective. Furthermore, the ICI-182,780-sensitive neuroprotection provided by T does not accommodate a nonspecific cholesterol-mediated component. In this model, cholesterol may not promote neuronal survival by any intrinsic activity of its own, but may be serving as a synthesis precursor for an explicitly protective steroid, e.g., 17β-E_2_. Others have also shown increased bioavailability of cholesterol to interact with effects mediated by steroids in the brain [50,51]. Evidence for all the necessary enzymes of synthesis in human brain has been reported [52,53] except for one, which has been identified in rat brain [54]. Because we use primary cell cultures derived from human fetal brain, it is likely that the metabolic machinery is present, operational and facilitating cholesterol's neuroprotection through an estrogenic end product. In support of our findings, neuroprotection by locally synthesized estrogen has recently been demonstrated [55], which when considered with the current findings, suggests possibilities for steroid prodrugs serving as neuroprotective therapeutics. However, others have demonstrated that cholesterol significantly reduces the neurotoxicity of gp120 by modulating membrane fluidity. In human neuroblastoma cells enriching cholesterol significantly reduced gp120 binding and consequently, the biochemical events triggered by the viral protein leading to necrotic death, while depleting cholesterol had the opposite effect [56]. Therefore the physiochemical properties of the membrane in mediating neurotoxicity/protection may be as significant as some pharmacological agents.

Synergistic neurotoxicity of the HIV proteins gp120 and Tat with cocaine was demonstrated in each experiment. Although neither the HIV protein combination nor cocaine alone was more neurotoxic than the Locke's buffer media, the simultaneous incubation of cells with both the HIV proteins and cocaine produced significant neuronal death. The mechanism by which cocaine facilitates the HIV protein toxicity is not well characterized. Recombinant variants of Tat protein (Tat 1–72 and Tat 1–86) are known to induce neuronal degeneration by mechanisms that involve triggering of apoptotic cascades [57,58]. Cocaine was recently shown to enhance gp120 induced neuronal apoptosis *in vivo *via a mechanism implicating glutamate excitotoxicity and iNOS expression with abnormal NO production in the neocortex of rat [59]. *In vitro *work in our lab with rat hippocampal cells supports a similar mechanism for cocaine's enhancement of Tat mediated toxicity by augmentation of ROS production and mitochondrial depolarization [60]. Likewise cocaine at very high concentrations *in vitro *may induce neuronal apoptosis [61]. Accordingly, estrogen neuroprotection could be via stimulation of the expression of anti-apoptotic proteins [62,63], or alternatively suppression of pro-apoptotic gene transcripts [21,64], although both of these interesting possibilities await further experimentation.

## Conclusion

In conclusion, we demonstrated that T provides neuroprotection through an ICI-182,708-sensitive mechanism against the synergistic toxicity of gp120 and Tat HIV proteins with cocaine. Additionally, we show that PROG, DHT and cholesterol provide some less efficacious neuroprotection than do the steroids, 17β-E_2 _and T. It is conceivable that gonadal steroids may provide biologically based gender-specific prophylaxis and/or amelioration either in the time of onset or symptomology of HAD and drugs of abuse. These results have implications for the innovation of therapeutic strategies, including steroid based neuroprotective prodrugs. Moreover, given that the neuropathology of HAD may have commonalities with dementias of other etiologies, the development of successful pharmacology for HAD would surely have applications for other equally devastating and costly neurodegenerative conditions.

## Methods

### Culture of human brain cells

Neuronal cultures were prepared as described previously [65-67]. Human fetal brain specimens of gestational age 12–14 weeks were obtained with the consent of women opting for elective termination of pregnancy. Protocols were carried out with strict adherence to the guidelines of the National Institutes of Health (NIH) and with approval from the University of Kentucky Institutional Review Board. Briefly, after mechanical dissociation, the cells were suspended in Opti-MEM with 5% heat-inactivated fetal bovine serum, 0.2% N_2 _supplement and 1% antibiotic solution (penicillin G sodium 10^4 ^units/ml, streptomycin sulfate10 μg/ml and amphotericin-B 25 μg/ml) (GIBCO). The cells were maintained in culture flasks at 37°C in a humidified atmosphere of 5% CO_2 _and 95% air for at least a month. At least 3 days before the experiment was performed the cells were plated in flat bottom 96 well plates coated with poly-*d*-lysine. Sample cultures were stained for the neuron-specific enolase, microtubule-associated protein-2, synaptophysin, and glial fibrillary astrocytic protein (GFAP). The cultures were thus characterized as more than 80% homogenous for neurons. The remaining cells were predominantly astrocytes (GFAP positive) with less than 1% microglia/macrophages, as determined by RCA-1 lectin and CD68 staining [67-69]-. Sample cultures were further characterized for the presence of ER-α and ER-β and dopaminergic neurons. As described earlier, ERs were visualized in 5–10% of cultured astrocytes and neurons in the cytoplasm and nuclei of both by immunostaining. Antigenicity for dopamine and dopamine transporter were co-localized in nearly 60% of neurons. Dopamine receptor D1A was seen in 50% of cells while receptor D2 was seen in 40% of cells. ER co-localized with cells staining for dopamine as well as D1A and D2 receptor containing neurons [30].

### Recombinant Tat and gp120 proteins

Recombinant Tat was prepared as described previously [67,70] with minor modifications. The *tat *gene encoding the first 72 amino acids was amplified from HIV_BRU _obtained from Dr. Richard Gaynor through the AIDS repository at the NIH and inserted into an *E coli *vector PinPoint Xa-2 (Promega). This construct allowed the expression the Tat1-72 protein as a fusion protein naturally biotinylated at the N-terminus. The biotinylated Tat protein was purified on a column of soft release avidin resin, cleaved from the fusion protein using factor Xa, eluted from the column, then desalted with a PD10 column. The Tat protein was further purified of endotoxin by passage through a polymycin B, cyanogen bromide, immobilized on cross-linked 4% beaded agarose column (Sigma). All purifications steps contained dithiothreitol to prevent oxidation of the proteins. Tat Protein was more than 95% pure as determined by SDS-PAGE followed by silver staining. Analysis by HPLC using a C4 column showed a single symmetrical peak. Western blot analysis showed that this preparation contained both monomeric and dimeric forms of Tat1-72. The functional activity of Tat1-72 was confirmed using a transactivation assay in HL3T1 cells containing an HIV-1 LTR-CAT construct. Chiron Corporation made a gift of gp120 from HIV_SF2 _which was described previously [30]. Briefly, recombinant gp120 was made in a Chinese Hamster Ovary cell line. Purification yielded 95% pure gp120 as determined by Western blot analysis. The Tat and gp120 preparations contained less than 1 pg/ml endotoxin as determined using a Pyrochrome Chromogenic test kit (Associates of Cape Cod, Inc., Falmouth, MA). The Tat proteins were stored in a lyophilized form and gp120 as a stock solution in water at -80°C in endotoxin-free siliconized microfuge tubes until taken for experimentation. Tat and gp120 are highly susceptible to degradation and loss of biological activity with each freeze-thaw cycle. Therefore, single aliquots were used for each experiment. Any remaining solution was discarded. Prior work has shown that no significant toxicity is seen with Tat less than 80 nM, gp120 less than 40 pM, or Tat (40 nM) plus gp120 (30 pM) [30]. For these studies, we used subtoxic dosage levels of Tat1-72 at 40 nM and gp120 at 32.5 pM.

### Drug levels

Cocaine hydrochloride obtained from the NIDA Drug Supply Program was solvated in Locke's buffer (pH 7.2) immediately prior to use. The dosage of cocaine (500 ng/ml) in culture translates to 1.6 μM, which is several orders of magnitude less than studies demonstrating a direct cytotoxic effect in culture [71-73]-. Fetal brain levels of cocaine of 1750 +/- 250 ng/ml were associated with fetal plasma levels of 500 ng/ml following repeated daily dosing in rat [74]. The plasma levels of cocaine from fetuses of mothers exposed to cocaine using the rat IV model [75] was 500 ng/ml as this represents 1/5 the peak brain levels in the fetus. This is also a concentration within the physiological levels experienced by human IV cocaine drug users [76]. Thus 500 ng/ml (1.6 μM) represents a physiologically relevant dose of cocaine to the neurons. Cocaine levels as high as 16 μM are not neurotoxic in human fetal neuronal cultures [30]. In these studies the cocaine solution was diluted such that addition of 1:10 (v/v) to the culture dishes resulted in a final concentration of 1.6 μM. This dose of cocaine was added concurrently with the Tat and gp120 proteins to the cultures.

### Neurotoxicity assay

At the time of the experimental treatment, the culture media was replaced with Locke's buffer containing (in mM) 154 NaCl, 5.6 KCl, 2.3 CaCl_2_, 1 MgCl_2_, 3.6 NaHCO_3_, 5 glucose, 5 N-2-hydroxyethylpiperazine-N'2-ethanesulfonic acid (HEPES) and 1% antibiotic solution (penicillin G sodium 10^4 ^units/ml, streptomycin sulfate10 μg/ml and amphotericin-B 25 μg/ml) (pH 7.2). Cells were incubated for 15 hours in Locke's buffer or with subtoxic concentrations of the HIV proteins, Tat1-72 (40 nM) plus gp120 (32.5 pM) [30], or cocaine (1.6 μM), or the HIV proteins plus cocaine to demonstrate the synergistic effect on cell death of HIV proteins with cocaine. To replicate the stereoisomer specific neuroprotective properties of estrogen, the cultures were treated with either 17β-E_2 _(10 nM), or 17α-E_2 _(10 nM) immediately preceding addition of the HIV proteins plus cocaine. To investigate the neuroprotective properties of other gonadal steroids, the cells were treated with various concentrations of PROG or T, immediately followed by exposure to the HIV proteins plus cocaine. To determine the mechanism of T's neuroprotection, the cultures were treated with various concentrations of DHT (5α-Androstan-17β-ol-3-one; 17β-Hydroxy-5α-androstan-3-one), or ICI-182,780 (100 nM) plus T (10 nM) before the HIV proteins plus cocaine were added. Various concentrations of cholesterol were used as treatment prior to incubation with the HIV proteins plus cocaine to further characterize the nature of gonadal steroid neuroprotection. ICI-182,780, DMSO (10 nM), β-cyclodextrin (100 nM) and ethanol (10 nM) were also tested for neurotoxic/protective effects. ICI-182,780 and 17α-E_2 _were solvated in DMSO. 17β-E_2_, T, PROG and cholesterol were β-cyclodextrin encapsulated to provide water solubility. Ethanol was used to solvate DHT. Tat1-72 was produced as indicated above, whereas gp120 was a gift from Chiron Corporation. The ICI-182,780 compound was obtained from Tocris Cookson, Inc. (Ellisville, MO). All other chemical agents were supplied by Sigma Chemicals (St. Louis, MO). At the end of the incubation period neuronal death was assessed by trypan blue (Sigma) exclusion assay as described previously [42,65,66]. In brief, each experiment was conducted in triplicate and at least three independent squads were treated with each pharmacological agent. Five randomly selected fields from each well were photographed using an inverted microscope (Nikon Diphot, 40X) and coded by a technician blinded to the well treatments. Viable and dead neurons in each photomicrograph were counted before decoding for statistical analysis. In representative fields the neurotoxicity assay was verified by false color visualization using the MCID digital camera-based system interfaced with a computer (Imaging Research, Ontario, Canada).

The neuronal and glial phenotypes seen in these cultures have been well characterized immunohistochemically and morphologically in our hands [65,66] as well as by others [77-79]-. Furthermore, the glia readily attach to the surface of the culture dish, whereas neurons do not [80]. In our hands, astrocytic attachment to the culture dish is requisite for the survival of the neurons, which in turn, attach to the substrate of glia. In this way, the glia are seen in a specific plane of microscopy that differs from the microscopic plane in which the neurons are seen. Therefore, we are confident of our ability to visually recognize and enumerate neurons as distinct from glia in these cultures.

### Analysis

Data are expressed as percent neuronal cell death/total viable neurons (means ± SEM). A 2 (presence or absence of the viral proteins gp120 and Tat) × 2 (presence or absence of cocaine) analysis of variance (ANOVA) design was employed to ascertain the potential for synergistic viral protein/cocaine-induced neurotoxicity. A significant interaction of the HIV proteins with Coc, as graphically illustrated by the lines diverging from parallel, provided evidence of toxic synergism. One-way ANOVAs were used to assess the neuroprotective potential of the various gonadal steroid pre-treatments. Regression analysis was used to determine concentration-effect relationships [81]. Planned Tukey-Kramer comparisons were used to determine specific treatment effects. An α level of p < 0.05 was considered significant for all statistical tests employed. Computer assisted analyses utilized BMDP Statistical Software, Release 7, Los Angeles, CA (1993) and SPSS Statistical Software release 11.5.0, SPSS, Inc., Chicago, IL (2004).

## Authors' contributions

SLK participated in the conception and design of the study, carried out the cell culture studies, quantified and analyzed the data, and drafted the manuscript. CFA participated in the design and coordination of the study. AN participated in the conception and design of the study and manuscript preparation. JT-C characterized the cell cultures and participated in manuscript preparation. CLL helped interpret the data and contributed to the preparation of the manuscript. CFM performed statistical and graphical analyses and helped draft the manuscript. RMB participated in the conception and design of the study and manuscript preparation and was the primary mentor of SLK. All authors read and approved the final manuscript.
